# COVID-19 Pandemic: Effect of Different Face Masks on Self-Perceived Dry Mouth and Halitosis

**DOI:** 10.3390/ijerph18179180

**Published:** 2021-08-31

**Authors:** Philipp Kanzow, Viktoria Dylla, Alannah Malina Mahler, Valentina Hrasky, Tina Rödig, Felix Barre, Simone Scheithauer, Annette Wiegand

**Affiliations:** 1 Department of Preventive Dentistry, Periodontology and Cariology, University Medical Center Göttingen, 37075 Göttingen, Germany; viktoria.dylla@stud.uni-goettingen.de (V.D.); alannahmalina.mahler@med.uni-goettingen.de (A.M.M.); valentinahrasky@med.uni-goettingen.de (V.H.); troedig@med.uni-goettingen.de (T.R.); annette.wiegand@med.uni-goettingen.de (A.W.); 2 Institute of Infection Control and Infectious Diseases, University Medical Center Göttingen, 37075 Göttingen, Germany; felix.barre@med.uni-goettingen.de (F.B.); simone.scheithauer@med.uni-goettingen.de (S.S.C.)

**Keywords:** COVID-19, dry mouth, face masks, halitosis

## Abstract

Due to the COVID-19 pandemic, the use of face masks has increased, resulting in potential health-related side-effects. Therefore, the study aimed to analyse the effect of wearing face masks on self-perceived dry mouth and halitosis. A questionnaire addressing the daily wearing time of different face masks (community masks, surgical/medical masks and KN95-/N95-/FFP2-masks) and self-perceived dry mouth and halitosis was given to adults attending or working at a university hospital. Statistical analysis was performed using Wilcoxon signed-rank test and multiple linear regression analysis (*p* < 0.05). 3750 participants (age: 50.4 ± 15.5 years; 60.0% female) were included. During the pandemic, face masks were used for 4.7 ± 3.8 h per day: community masks: 0.9 ± 2.0 h, medical/surgical masks: 1.9 ± 2.8 h and KN95-/N95-/FFP2-masks: 1.9 ± 2.5 h per day. The use of face masks significantly increased self-perceived dry mouth and halitosis (both *p* < 0.001). Self-perceived dry mouth and halitosis increased with increasing wearing time (community masks: dry mouth: *p* < 0.001, halitosis: *p* = 0.014; medical/surgical masks: both: *p* < 0.001; KN95-/N95-/FFP2-masks: dry mouth: *p* < 0.001, halitosis: *p* = 0.011). The perception of dry mouth and halitosis was increased in females compared to males (both: *p* < 0.001). Participants used to wearing face masks prior to the pandemic perceived dry mouth to a higher extent (*p* = 0.043). Self-perceived halitosis was lower in older than in younger participants (*p* < 0.001). Due to the increased perception of dry mouth and halitosis, people might abstain from wearing face masks. Further studies need to analyse measurable changes in dry mouth or halitosis.

## 1. Introduction

Face masks are used to prevent the severe acute respiratory syndrome coronavirus type 2 (SARS-CoV-2) transmission in healthcare facilities and public settings. The pandemic has resulted in a large increase in wearing face masks and the universal masking of health workers [1] and the public [2]. However, different face masks are used: According to current guidance issued by the World Health Organization (WHO), non-medical community masks are recommended for the public, while medical masks should be used by health workers in clinical areas. Particular respirator masks (KN95, N95, FFP2) should be used in clinical settings where aerosol-generating procedures are performed [3].

Potential health-related side-effects of wearing face masks are discussed. Recent systematic reviews addressed potential side effects and discomforts concerning the use of community masks, medical/surgical masks and FFP2/N95-masks with regard to a risk-benefit analysis [4,5]. Kisielinski et al. [5] found a significant effect of face masks, especially N95-masks, on the blood-oxygen depletion and symptoms of fatigue. The use of masks was also associated with headache, exertion, concentration problems and breathing difficulties [4,5]. Wearing N95-masks was also reported to increase facial skin temperature, humidity, heat, breathing difficulty and general discomfort compared to surgical masks [5,6].

So far, only limited information regarding potential oral side effects caused by face masks is available. In a questionnaire survey on the effects of wearing surgical masks or N95-masks for a minimum of 4 h per day, health care workers frequently reported nasal and skin symptoms, and to a lesser extent also dry mouth or halitosis [7]. Purushothaman et al. [7] point out that discomfort while wearing face masks might result in poorer adherence leading to an increased risk of infection. To limit discomfort and oral side-effects, the authors suggest nasal breathing while wearing masks. Another study reported dry mouth to belong to the most common problems when using face masks (N95-mask: 55.6%, surgical mask: 50.2%). Wearing N95 or surgical masks for more than 4 h significantly increased the risk of dry mouth [8]. Therefore, Atay and Cura [8] suggest shorter shifts for health care workers to limit health-related side-effects of face masks.

However, most of the studies were performed in a comparatively small cohort of healthcare professionals [7,8], but not in the general population. Moreover, the effect of different masks has not been analysed so far. Therefore, this questionnaire study aimed to analyse the effect of wearing time of face masks on self-perceived dry mouth and halitosis in a general population including healthcare professionals. The null hypotheses were that wearing a face mask does not increase self-perceived dry mouth and halitosis and that the wearing time of different masks (community mask, medical/surgical mask, KN95-/N95-/FFP2-mask) has no significant effect.

## 2. Materials and Methods

The study was approved by the local ethics committee of the University Medical Center Göttingen (no. 28/10/20) and registered at https://clinicaltrials.gov/ (NCT04632004) prior to its initiation.

The questionnaire was developed and reviewed by two dentists and two infection control scientists and composed of two parts: Part 1 contained questions regarding personal characteristics (age, gender), the wearing time per day of different face masks (1. community mask, 2. medical/surgical mask, 3. KN95-/N95-/FFP2-mask) and if face masks were worn before the COVID-19 pandemic (health care professionals and medical/dental students). Part 2 consisted of two item batteries with eight closed-ended questions regarding self-perceived dry mouth and halitosis without wearing a face mask (a) or while/directly after wearing a face mask (b). For both dry mouth and halitosis, a 5-point Likert scale ranging from 1 (never) to 5 (very often) was used.

Self-perceived dry mouth was assessed by five questions based on an adaptation of the short version of the Xerostomia Inventory [9]. Self-reported halitosis was addressed by three questions based on previous questionnaires [10,11]: While/directly after wearing a face mask/without wearing a face mask, …

1 I feel a bad taste in my mouth.
2 I perceive that I have bad breath.
3 I was told that I have bad breath.

The paper-based questionnaire was provided to patients, caregivers, health care workers, students, administrative staff and visitors (as far as allowed during the COVID-19 pandemic) of the University Medical Center Göttingen between November 2020 and June 2021. Participants were contacted face-to-face and invited to participate by random sampling. Participation in the survey was voluntary, and no incentives were given. Participants gave informed consent by returning the questionnaire in a ballot box.

Data from questionnaires was entered into a digital spreadsheet. Subsequently, statistical analysis was performed using the software R (version 4.1.0, www.r-project.org). Participants’ responses on each question with and without wearing face masks were compared using Wilcoxon signed-rank tests and *p*-values were adjusted according to Bonferroni–Holm. Dry mouth and halitosis with and without wearing face masks were also assessed using summated scores and compared using Wilcoxon signed-rank tests. The correlation between the effect of wearing face masks on self-perceived dry mouth and halitosis (Δ with and without wearing face masks) was evaluated using Spearman’s rank correlation. The effect of age, gender (female, male, third gender), wearing face masks pre-COVID-19 (yes, no) and wearing time of different face masks (community mask, surgical/medical mask and KN95-/N95-/FFP2-mask) on dry mouth and halitosis (Δ with and without wearing face masks) was assessed using multiple linear regression analyses.

## 3. Results

A total of n = 3750 questionnaires was returned (females: n = 2249, males: n = 1494, third gender: n = 7). Participants were aged 18 to 91 years (50.4 ± 15.5 years). Among all participants, n = 678 (18.1%) indicated that face masks were frequently worn before the COVID-19 pandemic (i.e., health care professionals and medical/dental students).

During the pandemic, participants used face masks for 4.7 ± 3.8 h per day. On average, community masks were worn for 0.9 ± 2.0 h per day, medical/surgical masks for 1.9 ± 2.8 h per day and KN95-/N95-/FFP2-masks for 1.9 ± 2.5 h per day.

Wearing face masks resulted in significantly increased self-perceived dry mouth (9.34 ± 3.77 vs. 6.74 ± 2.39, *p* < 0.001) and halitosis (5.68 ± 2.52 vs. 4.11 ± 1.54, *p* < 0.001). Self-perceived dry mouth and halitosis were significantly correlated (*p* < 0.001, ρ = 0.464). Detailed results regarding self-reported dry mouth and halitosis at the level of individual items are shown in Table 1.

Results from multiple linear regression analysis regarding the effects on the summated dry mouth score (Δ) are shown in Table 2. Self-perceived dry mouth increased with increased wearing time of community masks (*p* < 0.001, b = 0.101), medical/surgical masks (*p* < 0.001, b = 0.166) and KN95-/N95-/FFP2-masks (*p* < 0.001, b = 0.127). The perception of dry mouth was also increased in females compared to males (*p* < 0.001, b = 0.605) and in participants being used to wearing face masks from pre-COVID-19 (*p* = 0.043, b = 0.282).

Results from multiple linear regression analysis regarding the effects on the summated halitosis score (Δ) are shown in Table 3. Self-perceived halitosis increased with increased wearing time of community masks (halitosis: *p* = 0.014, b = 0.043), medical/surgical masks (*p* < 0.001, b = 0.067) and KN95-/N95-/FFP2-masks (*p* = 0.011, b = 0.038). The perception of halitosis was also increased in females compared to males (*p* < 0.001, b = 0.341). The perception of halitosis was lower in older than in younger participants (*p* < 0.001; b = −0.019).

## 4. Discussion

The identification of potential subjective or objective side effects of protective measures is highly relevant, i.e., to understand the adherence to protective measures (here: face masks) during the SARS-CoV-2 pandemic. For example, potential health-related side-effects of wearing face masks are within the top five topics associated with the public discourse against wearing face masks during the pandemic [12].

This study showed that wearing face masks increases the self-perception of dry mouth and halitosis, and that wearing time of different masks affects self-perception. Thus, both null hypotheses must be rejected. Results are in line with previous studies which also found face masks to impact on self-perceived dry mouth [7,8] and halitosis [7,13,14].

Largest differences were observed between the items “mouth feels dry” and “lips feel dry”, most probably as wearing face masks is likely to change the breathing pattern. However, airflow and breathing resistance vary between different face masks based on mask material and proportion of leakage: KN95-/FFP2-masks and community masks were reported to result in higher breathing resistance than medical face masks, leading to potential leakage [15]. Dry mouth is most probably perceived due to a change of the breathing pattern from nasal to oral breathing while wearing masks [16]. Mouth breathing increases the subjective perception of dry mouth [17], although most studies found no measurable difference in salivary flow of patients with nasal or oral breathing [18,19]. Evaporation during mouth breathing might also enable the release of volatile sulphur compounds into the mouth air leading to halitosis [20,21]. Wearing N95-masks leads to higher levels of self-perceived humidity than wearing surgical masks [6]. Thereby, wearing surgical/medical masks might result in increased evaporation. This might explain why wearing time of surgical/medical masks more strongly impacted on the change in summated dry mouth and halitosis scores than wearing other masks.

Within the present study, 13.3% answered one or multiple items regarding halitosis with at least “sometimes” while not wearing a face mask. A previous study reported the prevalence of self-perceived halitosis among German adults to amount to 20.1% [22]. Dry mouth and halitosis are closely related [23] and associated with reduced oral health-related quality of life [24,25]. Regarding the use of face masks, this study found that self-perceived dry mouth and halitosis are significantly correlated. This is in line with a previous study which identified dry mouth as a significant risk factor for self-perceived halitosis [14]. Confirming previous studies, both conditions were more prevalent in females compared to males [26,27]. Also, the perception of dry mouth increased with increasing age [26], while the opposite was observed for halitosis.

Interestingly, differences between health care professionals and people from the general population were observed: Participants that were adapted to the frequent use of face masks, thus professional health care workers and medical/dental students, perceived dry mouth and halitosis to a higher extent than people from the general population. Most probably this observation can be explained by an increased knowledge and awareness for (oral) health by health care professionals compared to the general population [28,29].

This study was conducted in a large cohort of people working at or attending a university hospital in Germany. Due to the research design (i.e., contacting potential participants in-person), calculation of a response rate was impossible. However, demographic data of the participants well represents the overall demographics of the German population: On average, German adults are 51.2 years old and the gender ratio amounts to 1:1 [30]. The higher proportion of females within the present study might be attributed to the participation of healthcare professionals and medical/dental students: About 18% of the participants were adapted to the use of face masks already before the pandemic, indicating that these participants are professional health care workers or medical/dental students. This proportion seems reasonable as adults inside a university hospital were invited by random sampling. Among German health care professionals, a higher proportion of females (75.5%) exists [30]. Also, the proportion of female medical/dental students at the specific university hospital amounts to 64.9% [31].

The results of the present questionnaire survey present the self-perceived change in oral health of health care professionals and people from the general population. Self-perceived dry mouth was assessed based on an adaptation of the short version of the Xerostomia Inventory, which is a validated tool for assessing dry mouth [9]. For assessing halitosis, questions were adapted from previous questionnaire surveys [10,11]. The questionnaire was kept short to increase the willingness to participate in the survey. As a consequence, the study does not allow for drawing conclusions regarding the preferred use of a specific face mask as the overall effect of different masks on dry mouth and halitosis was assessed. Instead of using a 5-point Likert scale, a visual analogue scale might have yielded more detailed results. Also, further factors with potential impact on self-perceived dry mouth and halitosis (e.g., drinking habits, oral hygiene measures) were not assessed. Moreover, the regulations in Germany changed during the study period: From February 2021 onwards, community masks were no longer allowed when entering the University Medical Center Göttingen. Instead, medical/surgical masks or KN95-/N95-/FFP2-masks became mandatory [32].

## 5. Conclusions

The use of face masks increased the perception of dry mouth and halitosis. Further studies need to analyse whether the use of face masks lead to a measurable change in dry mouth or halitosis (i.e., by measuring the salivary flow rate and volatile sulphur compounds).

## Figures and Tables

**Table 1:** Descriptive results regarding self-reported dry mouth and halitosis.

Item	Face Masks	Mean	SD	*p*-Value
mouth feels dry	with	2.53	1.25	**<0.001**
	without	1.49	0.73	
lips feel dry	with	2.62	1.28	**<0.001**
	without	1.72	0.87	
mouth feels dry when eating a meal	with	1.54	0.87	**<0.001**
	without	1.21	0.51	
problems eating dry foods	with	1.42	0.81	**<0.001**
	without	1.20	0.54	
difficulty in eating certain foods	with	1.25	0.65	**<0.001**
	without	1.14	0.47	
bad taste in mouth	with	2.02	1.18	**<0.001**
	without	1.43	0.66	
perceive bad breath	with	2.44	1.28	**<0.001**
	without	1.52	0.74	
told to have bad breath	with	1.23	0.63	**<0.001**
	without	1.17	0.47	

A 5-point Likert scale ranging from 1 (never) to 5 (very often) was used. Self-perceived dry mouth was assessed by the first five questions based on an adaptation of the short version of the Xerostomia Inventory [9]. All *p*-values were adjusted according to Bonferroni–Holm. Significant *p*-values are printed in bold. SD: Standard deviation.

**Table 2:** Descriptive statistics of single parameters regarding the change in summated dry mouth score (mean ± standard deviation) and effect estimates, 95% confidence intervals and *p*-values from multiple linear regression analysis.

Parameter	Level	Score Δ_[with-without]_	Estimate [b]	95% Confidence Interval	*p*-Value
(intercept)			1.625	1.201–2.049	**<0.001**
age [years]			−0.002	−0.009–0.005	0.590
gender	male	2.14 ± 2.96			
	female	2.89 ± 3.26	0.605	0.398–0.812	**<0.001**
	third gender	1.14 ± 2.27	−1.117	−3.416–1.181	0.341
wearing face mask pre-COVID-19 [h]	no	2.47 ± 3.07			
	yes	3.16 ± 3.49	0.282	0.009–0.555	**0.043**
wearing time community mask [h]			0.101	0.050–0.153	**<0.001**
wearing time surgical/medical mask [h]			0.166	0.126–0.205	**<0.001**
wearing time KN95-/N95-/FFP2-mask [h]			0.127	0.084–0.170	**<0.001**

Δ_[with-without]_: Change in summated dry mouth score with and without wearing face masks. Significant *p*-values are printed in bold.

**Table 3:** Descriptive statistics of single parameters regarding the change in summated halitosis score (mean ± standard deviation) and effect estimates, 95% confidence intervals and *p*-values from multiple linear regression analysis.

Parameter	Level	Score Δ_[with−without]_	Estimate [b]	95% Confidence Interval	*p*−Value
(intercept)			2.039	1.751–2.327	**<0.001**
age [years]			−0.019	−0.023–−0.014	**<0.001**
gender	male	1.32 ± 2.02			
	female	1.73 ± 2.22	0.341	0.200–0.481	**<0.001**
	third gender	0.86 ± 2.04	−0.822	−2.388–0.743	0.303
wearing face mask pre-COVID-19	no	1.50 ± 2.07			
	yes	1.88 ± 2.45	0.168	−0.016–0.352	0.074
wearing time community mask [h]			0.043	0.009–0.078	**0.014**
wearing time surgical/medical mask [h]			0.067	0.040–0.093	**<0.001**
wearing time KN95-/N95-/FFP2-mask [h]			0.038	0.009–0.067	**0.011**

Δ_[with-without]_: Change in summated halitosis score with and without wearing face masks. Significant *p*-values are printed in bold.

## Data Availability

The data presented in this study are available on request from the corresponding author. The data are not publicly available due to ethical and privacy issues.
